# Supplementary material for: Resolving the full spectrum of human genome variation using Linked-Reads
Source: Genome Res. 2019 Apr;29(4):635–45. doi: 10.1101/gr.234443.118 (PMC6442396; doi:10.1101/gr.234443.118)
Supplement: Supplemental Material [file supp_gr.234443.118_Supplemental_longranger-2.2.zip › longranger-2.2.tar.bz2!/lr2.2-public/lib/stan/cmdstan/stan/doc/api/html/agrad_2fwd_2matrix_2log__sum__exp_8hpp_source.html]

Stan: src/stan/agrad/fwd/matrix/log\_sum\_exp.hpp Source File


|  |  |
| --- | --- |
|  | Stan  2.5.0  probability, sampling & optimization |


- Main Page
- Related Pages
- Namespaces
- Classes
- Files

- File List
- File Members

All Classes Namespaces Files Functions Variables Typedefs Enumerations Enumerator Friends Macros Pages

- src
- stan
- agrad
- fwd
- matrix

log\_sum\_exp.hpp

Go to the documentation of this file.

1 #ifndef STAN\_\_AGRAD\_\_FWD\_\_MATRIX\_\_LOG\_SUM\_EXP\_HPP

2 #define STAN\_\_AGRAD\_\_FWD\_\_MATRIX\_\_LOG\_SUM\_EXP\_HPP

3

4 #include <vector>

5 #include <stan/agrad/fwd/fvar.hpp>

6 #include <stan/math/matrix/log\_sum\_exp.hpp>

7 #include <stan/math/matrix/Eigen.hpp>

8 #include <stan/agrad/fwd/functions/log.hpp>

9 #include <stan/agrad/fwd/functions/exp.hpp>

10

11 namespace stan{

12

13  namespace agrad{

14

15  // FIXME: cut-and-paste from fwd/log\_sum\_exp.hpp; should

16  // be able to generalize

17  template <typename T, int R, int C>

18  fvar<T>

19  log\_sum\_exp(const Eigen::Matrix<fvar<T>,R,C>& v) {

20  using stan::math::log\_sum\_exp;

21  using stan::agrad::log\_sum\_exp;

22  using stan::agrad::exp;

23  using std::exp;

24  using stan::agrad::log;

25  using std::log;

26

27  Eigen::Matrix<T,1,Eigen::Dynamic> vals(v.size());

28  for (int i = 0; i < v.size(); ++i)

29  vals[i] = v(i).val\_;

30  T deriv(0.0);

31  T denominator(0.0);

32  for (size\_t i = 0; i < v.size(); ++i) {

33  T exp\_vi = exp(vals[i]);

34  denominator += exp\_vi;

35  deriv += v(i).d\_ \* exp\_vi;

36  }

37  return fvar<T>(log\_sum\_exp(vals), deriv / denominator);

38  }

39

40  }

41 }

42 #endif

stan::agrad::log\_sum\_exp

fvar< T > log\_sum\_exp(const fvar< T > &x1, const fvar< T > &x2)

**Definition:** log\_sum\_exp.hpp:15

stan::math::log\_sum\_exp

boost::math::tools::promote\_args< T1, T2 >::type log\_sum\_exp(const T2 &a, const T1 &b)

Calculates the log sum of exponetials without overflow.

**Definition:** log\_sum\_exp.hpp:49

stan::agrad::fvar

**Definition:** fvar.hpp:13

log.hpp

fvar.hpp

Eigen.hpp

exp.hpp

log\_sum\_exp.hpp

stan::agrad::log

fvar< T > log(const fvar< T > &x)

**Definition:** log.hpp:15

stan::agrad::exp

fvar< T > exp(const fvar< T > &x)

**Definition:** exp.hpp:16


---

[ Stan Home Page ]

*© 2011–2014,
Stan Development Team.*
